# Histopathological and Genomic Grading Provide Complementary Prognostic Information in Breast Cancer: A Study on Publicly Available Datasets

**DOI:** 10.4061/2011/890938

**Published:** 2011-08-10

**Authors:** Nilotpal Chowdhury

**Affiliations:** Department of Pathology, Aarupadai Veedu Medical College, Bahour Commune Panchayat Kirumampakkam, Pondicherry 607402, India

## Abstract

The genomic grade (GG) for breast cancer is thought to be the genomic counterpart of histopathological grade (HG). The motivation behind this study was to see whether HG retains its prognostic impact even when adjusted for GG, or whether it can be replaced by the latter. Four publicly available gene expression datasets were analyzed. Kaplan-Meier curves, log rank test, and Cox regression were used to study recurrence-free survival (RFS) and distant metastasis-free survival (DMFS). HG remained a significant prognostic indicator in low GG tumors (*P* = 0.003 for DMFS, *P*< 0.001 for RFS) but not in high GG tumors. HG grade 2 tumors differed significantly from HG grade 1 tumors, underlining the prognostic role of intermediate HG tumors. Additionally, GG could stratify HG 1 as well as HG 2 tumors into distinct prognostic groups. HG and GG add independent prognostic information to each other. However, the prognostic effects of both HG and GG are time varying, with the hazard ratios of high HG and GG tumors being markedly attenuated over time.

## 1. Introduction

The genomic grade [1] is a recently characterized grading system for breast cancer which has shown early promise in prognostic classification of breast cancer patients. It is a two-tier grading system which has appealed to researchers in doing away with an intermediate grade, ostensibly easing the decision-making process. Furthermore, it has a strong theoretical basis, since it is judged to be a gene expression signature of the histopathological grade, a proven independent predictor of breast cancer survival. The genomic grade has met with favorable academic response and has been licensed for commercial use. Early studies suggested that it contained most of the prognostic information of histopathological grade [1], thus leading to suggestions that it may supplant histopathological grading altogether [2, 3]. One study found that the genomic grade was a better prognostic indicator than histopathological grade [4]. However, it was not mentioned whether histopathological grade had any prognostic effect in breast cancer patients when adjusted for genomic grade. The datasets used in the last mentioned study were put in the public domain in an easily analyzable format. The same datasets were used in the present study to see whether histopathological grading provides any additional information over and above the genomic grade. This would serve to point out whether gene expression-based signatures could act as a competitive replacement for histopathological grade, or whether both genomic and histopathological grade are independently significant and complement each other.

## 2. Materials and Methods

An internet search was made for publicly available breast cancer datasets containing data on the histopathological grade, genomic grade, and survival. Four such datasets were found based on Affymetrix microarray platform, which were the basis for previously published papers [4].

A study on the four combined datasets was published [4] showing that a simple gene expression-based model could give prognostic information of the same or better quality than more complex models. Furthermore, the authors of the last mentioned article also put up the clinical data in convenient Rdata files on the web (http://www.ulb.ac.be/di/map/bhaibeka/survcompaper/) which immensely helped in the final analysis of the data. The same datasets also helped in the formation and validation of a fuzzy gene expression-based prognostic signature known as GENIUS [5].

 These datasets are publicly available from the GEO database through accession numbers GSE2034, GSE7390, GSE6532/GSE9195, and GSE3494, respectively. GSE2034 includes the gene expressions of 286 node-negative patients who did not receive adjuvant therapy, and was used to build the 76 gene expression signature [6]. It was also used as a validation dataset for the genomic grade. GSE7390 comprised node-negative patients who did not have any adjuvant therapy. It was used to demonstrate strong time dependence of prognostic effects of the 76 gene signature [7]. Additionally, it was an official validation dataset for the genomic grade index. GSE6532/9195 has 354 ER-positive node-negative as well as node-positive patients homogeneously treated by tamoxifen therapy and formed the basis for two papers [8, 9]. GSE3494 was a heterogeneous dataset of 251 patients and was used to study the genetic expression of p53 in breast cancer patients [10]. All the datasets had information for recurrence-free survival (RFS), but GSE 3494 was missing information on distant metastasis-free survival (DMFS). The four datasets were combined for the present analysis. The distribution of the clinicopathological parameters in the combined data is given in Table 1.

Kaplan-Meier survival curves, log-Rank test, and Cox proportional hazards regression were used to assess the prognostic role of histopathological grade in predicting RFS and DMFS in both low and high genomic grade cases. The Cox proportional Hazards regression model was assessed for fit. Nonproportionality of hazards was checked by scaled Schoenfield residuals. Influential observations were checked by plotting the dfbetas. 

The datasets were prepared from different series of patients from different centres. There were twelve separate series of patients, some of them having different selection criteria. Therefore, patients in one series could be more similar to another patient in the same series as compared to a patient in a different series (i.e., the data may be clustered along the series from which the patients were selected). To compensate for the effect of clustered data, a grouped jackknife method for estimation of robust parameter variances was used for the Cox proportional hazards analysis as recommended [11]. 

The entire analysis was carried out by the “survival” [12] package of the R statistical environment [13].

## 3. Results

**Figure 1:**
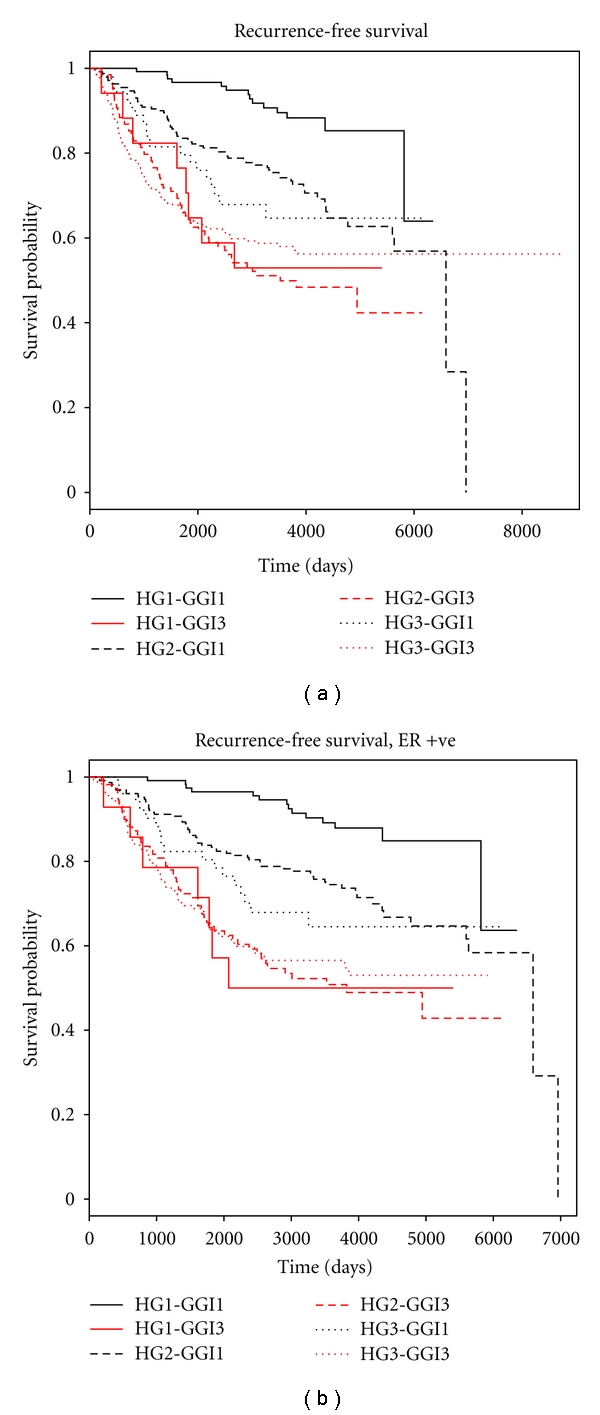
(a) Kaplan-Meier curve for recurrence-free survival for all the patients, showing the separation of survival curves between Grade 1, 2, and 3 low genomic grade patients and closeness of the same in high genomic grade patients. (HG = histopathological grade, GGI = genomic grade). (b) Kaplan-Meier curve for recurrence-free survival for ER-positive patients, showing the separation of survival curves between Grade 1, 2, and 3 low genomic grade patients and closeness of the same in high genomic grade patients. (HG = histopathological grade, GGI = genomic grade).

**Figure 2:**
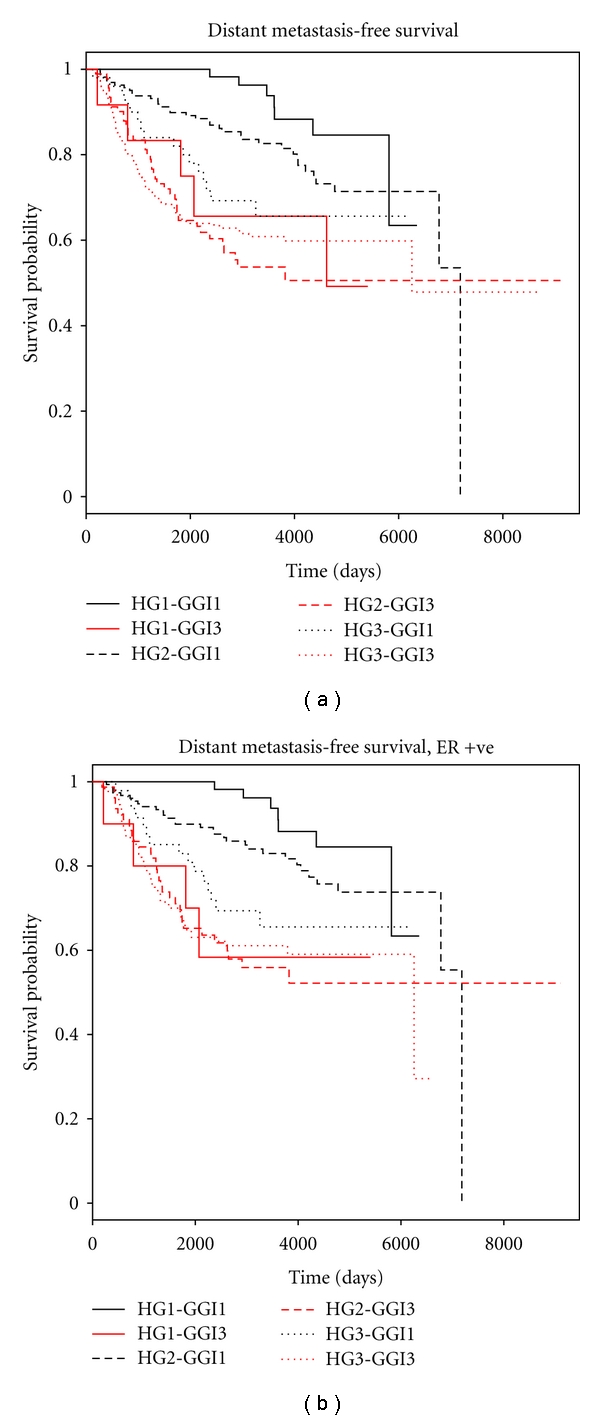
(a) Kaplan-Meier curve for distant metastasis-free survival for all the patients, showing the separation of survival curves between Grade 1, 2, and 3 low genomic grade patients and closeness of the same in high genomic grade patients. (HG = histopathological grade, GGI = genomic grade). (b) Kaplan-Meier curve for distant metastasis-free survival for ER-positive patients, showing the separation of survival curves between Grade 1, 2, and 3 low genomic grade patients and closeness of the same in high genomic grade patients. (HG = histopathological grade, GGI = genomic grade).

Histopathological grade was a significant predictor for survival in low genomic grade (GG1) patients, for the entire dataset (*P* = 0.003 for DMFS, *P* < 0.001 for RFS, by the log rank test) but not in high genomic grade (GG3) patients (*P* = 0.8 and 0.9 for DMFS and RFS, respectively, by the log rank test). Kaplan-Meier curves showed separation of the survival curves of low, intermediate, and high histopathological grade tumors in low genomic grade patients, but the separation between the same was not prominent in high genomic grade patients (Figures 1 and 2). Particular attention was paid to ER positive tumors, as it has been shown that the genomic grade is a particularly strong prognostic factor in ER positive [5] but not in ER negative patients. Further tests by Cox proportional hazards showed that the histopathological grade was a significant predictor in the entire set of ER-positive patients as well as node-negative, node-positive and Stage I ER-positive patients in low genomic grade tumors (Tables 2 and 3—compare the hazard ratios of HG3-GG1 and HG2-GG1 tumors vs. HG1-GG1 tumors). The continued prognostic difference between histopathological grade 2 and grade 1 patients in the low genomic grade tumors for RFS suggest that contrary to early expectations, grade 2 patients remain a distinct prognostic category.

Tables 2 and 3 also show that the genomic grade was a significant prognostic factor not just in histopathological grade 2 (*P* values given in Supplementary file, Table 2, which is available online at doi:10.4061/2011/890938) but also in histopathological grade 1 breast cancer patients. This shows that the genomic grade is useful not just in stratifying histopathological grade 2 cases but identifies a bad prognostic subset in histopathological grade 1 cases as well. Even histopathological grade 3 cases were separated into separate groups by the genomic grade having at least marginal statistical significance in certain groups (*P* values given Supplementary file, Table 2), but the prognosis was bad in histopathological grade 3 cases irrespective of the genomic grade status, limiting the clinical usefulness of genomic grade in the same. These results are illustrated in the Kaplan-Meier curves in Figures 1 and 2, where there is a wide prognostic separation between low and high genomic grade tumors for both histopathological grade 1 as well as histopathological grade 2 cases. However, there is a narrow separation between low and histopathological grade 3 cases having a low and high genomic grade in histopathological grade 3 tumors, with both sets having a poor prognosis. 

While checking the models by Schoenfeld residuals, a significant time-varying prognostic effect of histopathological grade and genomic grade was observed, with high histopathological and genomic grade tumors showing a strong prognostic effect initially, but becoming attenuated on the long term (Table 4). For further demonstration of the marked time-varying covariate effects, the data was partitioned at 7 years, and Cox regression was carried out separately in different time intervals (0–7 years, and 7 years to end of study). In the time interval from 0 to 7 years, both histopathological and genomic grade showed strong and statistically significant information; however, in the time interval from seven years to the end of the study, the hazard ratios were markedly reduced, and not a single combination of high genomic and histopathological grade showed statistically superior prognostic effect compared to the lowest grading category (Table 5). In fact, the highest combined grade showed a reversal of the hazard ratio over the lowest grading combination. It may be noted that for this particular analysis, time was partitioned at seven years just for illustrative purposes, and decreasing hazard ratios over time will be noted even if the study is partitioned at other time intervals. The time-varying prognostic effect of histopathological grade and genomic grade can also be deduced from the converging Kaplan-Meier curves for RFS and DMFS in Figures 1 and 2. This did not affect the conclusion in the present study that histopathological grade is an important factor in low genomic grade but not in high genomic grade patients. However, it is important to be aware of this property of breast cancer grade so that the attenuation of prognostic effect is not mistaken for absence of the same in studies with a long followup.

## 4. Discussion

The results in the study on this publicly available dataset emphasizes the continued importance of histopathological grade in breast cancer prognostication, even when adjusted for recently discovered genetic expression-based analogue of grading. This study confirms the prognostic importance of the intermediate histopathological grade and additionally suggests a prognostic role for genomic grade even in histopathological grade cases. Both histopathological and genomic grade add prognostic information to one another and should be seen as complementary rather than competitive techniques.

The “old fashioned” histopathological grade remains an important prognosticator in breast cancer in spite of the presence of newer gene expression grade. The importance of the old school indicators have been emphasized in other studies (comparing other gene expression-based profiles) as well. The supplementary information provided by Fan et al. [14] shows that the histopathological grade retains either prognostic significance or a high hazard ratio even when adjusted for different gene expression-based signatures like the Agendia 70 gene signature or the Oncotype Dx signature. Studies on the prognostic efficiency of the Oncotype Dx signature [15, 16] have repeatedly thrown upgrade as an independent prognostic factor in multivariate analysis. Recently, it was shown that clinical data inclusive of histopathological grade added significantly to the prognostic ability of gene expression signatures [17]. A review of the role of histopathological grading in the molecular era also concluded that gene expression signatures and histopathological grading play a complementary role in breast cancer prognostication [18]. The genomic grade, analyzed in this study, was obtained as the genetic analogue of histopathological grade, and thus was likely to be the most significant competitor to the latter; however, the additional prognostic information given by histopathological grade in this study shows that these techniques should be treated as complementary rather than competitive. The genomic grade was created using ER-positive tumors only [1] and does not have a strong prognostic effect in ER-negative tumors [5, 19]. Therefore, this study paid particular attention to ER-positive tumors in pointing out the independent prognostic role of histopathological grade. Histopathological grade retained its prognostic significance in ER-positive, ER-positive node negative, as well as ER-positive Stage I tumors, showing its prognostic strength independent of nodal status and size in addition to genomic grade.

The present study analyzes different datasets graded by different pathologists. It is possible that there may have been a loss in prognostic effect of histopathological grading due to interobserver variability between centres, but the continued prognostic significance of histopathological grade shows that it remains a strong prognostic factor in spite of the subjectivity of grading. In other words, the “signal” provided by the histopathological grade more than compensates for the “noise” due to interobserver variation. Centralized grading may have improved the observed prognostic strength of histopathologic grading even further, but it would not have reflected the “real-world” effect of histopathological grading, taking interobserver variation in account. The lack of interobserver reliability of histopathological grade is a constant criticism against its inclusion as a treatment deciding factor. In the light of strong evidence of the prognostic strength of the histopathological grading, steps should be taken to improve the interobserver reliability of the same rather than throw it out altogether. Strict adherence to the grading guidelines [18, 20] as well as double reporting [21] has been shown to result in high interobserver reproducibility of breast cancer histopathological grading. 

Intermediate histopathological grade tumors showed a significantly raised hazard ratio for recurrence-free survival in low genomic grade tumors, emphasizing the prognostic importance of the intermediate grading category. One of the potential attractions of the genomic grade was the absence of the intermediate category; it was hoped that this would help give a clearer picture of prognostic strength of breast cancers. The genomic grade challenged the existence of intermediate histological grade [3]. However, the continued importance of the intermediate category suggests that breast cancer grade is inherently a continuous prognostic indicator, something a surgical pathologist observing the microscopic sections will attest to. It should be noted that the present aggregated dataset contained the validation dataset of the genomic grade, which may bias the results against histopathological grade as a competitor to genomic grade. That the intermediate category still retains its prognostic significance testifies for the inherent prognostic strength of histopathological grading.

One of the main motivations behind the genomic grade was to classify histopathological grade 2 tumors into separate prognostic groups resembling grade 1 and grade 3 tumors [1]. However, the present study also showed that the genomic grade has a significant prognostic effect in histopathological grade 1 tumors, a finding which, to the best of the author's knowledge, has not been reported yet. This suggests that the genomic grade may have a wider clinical applicability in identifying bad prognosis tumors. Both low histopathological and genomic grade may be subclassified into heterogeneous prognostic categories. In contrast, high-grade tumors seem to be more prognostically homogeneous, and there is as yet no evidence for a clinically significant role of genomic grade in histopathological grade 3 tumors or histopathological grade in high genomic grade tumors. 

The prognostic strength of both histopathological as well as genomic grading is most marked in the short to mid-term, but lose their prognostic power for predicting late events. The loss of prognostic efficiency is a well-known attribute of intrinsic prognostic factors of breast cancer. Hormone receptor status, markers of proliferation, histopathological grade, size, and lymph node status [22, 23] all have been shown to lose their prognostic strength over time. There may be a few explanations for the loss of prognostic strength of known factors: (i) late survival events may depend on further genetic changes that take place after diagnosis as a result of the varied natural course of breast cancer, in which case the early genetic changes, as reflected in grade and proliferation at the time of diagnosis may not hold relevance for late events, (ii) the late events may be the result of subtle genetic changes as yet not discovered, or (iii) there is a process of “natural selection” of high grade cases, where the long-term survivors, who have as yet unknown favourable characteristics, survive. 

The analysis of the data was complicated by the presence of heterogeneity due to different selection criteria among the different constituent datasets, forming clustered data leading to a potential violation of the assumption of independence of cases in Cox regression. To overcome this complication, two types of models may have been used depending on the purpose of the study: (i) a marginal model or (ii) a frailty model [11]. In studies where the primary focus of interest lies in estimating the prognostic effects of covariates in the general population across clusters, and the relation between the correlated data is of little interest (as in this study), it is simpler to use a marginal model. In frailty models, the effects of the correlated clusters are modeled as a random effect along with the fixed effects of the other covariates of interest (forming what is known as a mixed effects model). Frailty models are used if there is interest in estimating the correlation between the clustered data. However, frailty models are complicated, and there are various frailty models depending on the assumed distributional properties of the random effect. For the reasons given above, marginal models were used in the present study.

The findings of this study need to be cautiously interpreted due to the large amounts of missing data. The data was not missing at random, and therefore, the final models may be biased. However, the consistently raised hazard ratio for histological grade in the analysis carried out in the component datasets suggests strongly that histopathological grade continues giving prognostic information over its genomic counterpart.

To conclude, both “old-fashioned” traditional and “modern” gene expression-based prognosticators give additional information over one another. These techniques need to coexist rather than compete for optimal patient management at the present time.

## Figures and Tables

**Figure 3:**
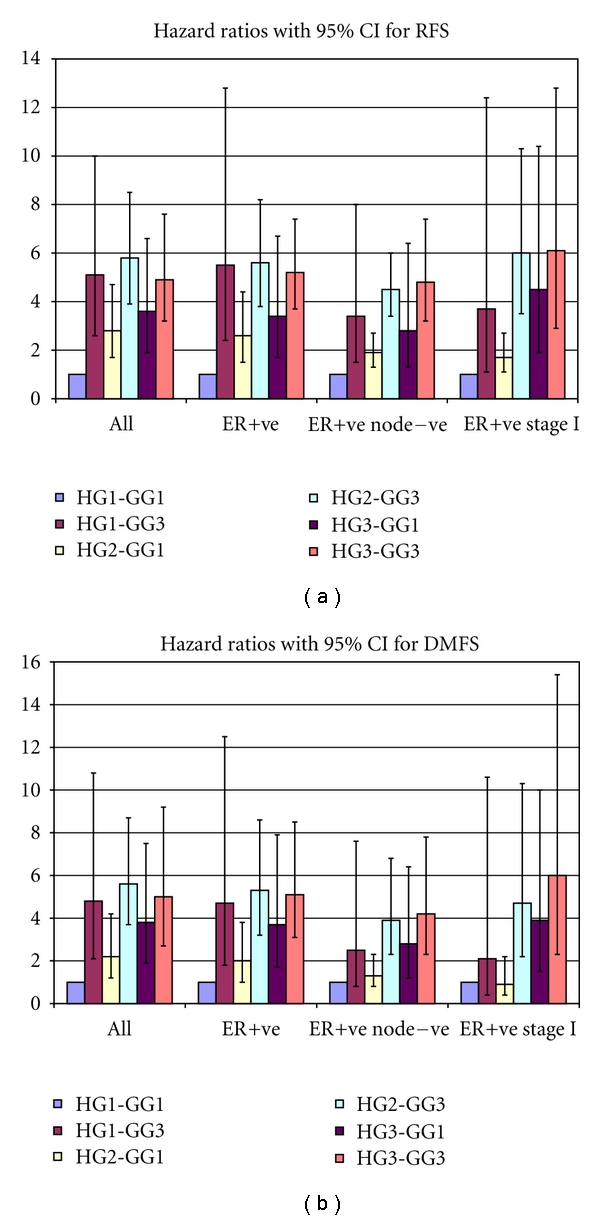
(a) and (b) showing the hazard ratios of the different combinations of histopathological grade and genomic grade for recurrence-free survival and distant metastasis-free survival. The solid columns represent the hazard ratios, and the lines with whiskers represent the 95% confidence intervals of the same.

**Table 1:** Clinicopathological features of all breast cancer patients analyzed in the study.

Age	
≤40 years	97
41–55 years	381
56–70 years	397
>70 years	205
Missing	9

Size	
<2 cm	520
>2 cm	560
Missing	9

Node	
Positive	799
Negative	263
Missing	27

ER	
Positive	896
Negative	180
Missing	13

Histopathological grade	
Grade 1	168
Grade 2	404
Grade 3	356
Missing	161

Genomic grade	
Low	501
High	488
Missing	100

Adjuvant treatment	
None	655
Tamoxifen	425
Missing	9

**Table 2:** Showing the hazard ratios for recurrence-free survival for each combination of histopathological and genomic grade. (HG = histopathological grade, GG1 = low genomic grade, and GG3 = high genomic grade). Values marked with an asterisk (*) are unstable due to scanty number of cases.

	All tumors		ER+ve tumors		ER+ve Node −ve tumors		ER+ve Stage I tumors		ER+ve Node +ve tumors	
	HR	95%CI *P*	HR	95%CI *P*	HR	95%CI *P*	HR	95%CI *P*	HR	95%CI *P*
HG1-GG3 versus HG1-GG1	5.1	2.6–10.0 (<0.001)	5.5	2.4–12.8 (<0.001)	3.4	1.5–8.0 (0.004)	3.7	1.1–12.4 (0.04)	19.6*	3.5–110* (<0.001)
HG2-GG1 versus HG1-GG1	2.8	1.7–4.7 (<0.001)	2.6	1.5–4.4 (<0.001)	1.9	1.3–2.7 (0.001)	1.7	1.1–2.7 (0.02)	6.8	1.4–33.2 (0.02)
HG2-GG3 versus HG1-GG1	5.8	3.9-8.5 (<0.001)	5.6	3.8-8.2 (<0.001)	4.5	3.4-6.0 (<0.001)	6.0	3.5-10.3 (<0.001)	11.4	2.8–46.9 (0.001)
HG3-GG1 versus HG1-GG1	3.6	1.9–6.6 (<0.001)	3.4	1.7–6.7 (<0.001)	2.8	1.3–6.4 (0.01)	4.5	1.9–10.4 (<0.001)	8.9*	2.0–39* (0.004)
HG3-GG3 versus HG1-GG1	4.9	3.2–7.6 (<0.001)	5.2	3.7–7.4 (<0.001)	4.8	3.2–7.4 (<0.001)	6.1	2.9–12.8 (<0.001)	7.9	2.3–27.7 (0.001)

**Table 3:** Showing the hazard ratios for distant metastasis-free survival for each combination of histopathological and genomic grade (HG = histopathological grade, GG1 = low genomic grade, and GG3 = high genomic grade). Values marked with an asterisk (*) are unstable due to scanty number of cases.

	All tumors		ER+ve tumors		ER+ve Node −ve tumors		ER+ve Stage I tumors		ER+ve Node +ve tumors	
	HR	95%CI *P*	HR	95%CI *P*	HR	95%CI *P*	HR	95%CI *P*	HR	95%CI *P*
HG1-GG3 versus HG1-GG1	4.8	2.1–10.8 (<0.001)	4.7	1.8–12.5 (0.002)	2.5	0.8–7.6 (0.1)	2.1	0.4–10.6 (0.4)	22.3*	3.1–162* (0.002)
HG2-GG1 versus HG1-GG1	2.2	1.2–4.2 (0.01)	2.0	1.04–3.8 (0.04)	1.3	0.8–2.3 (0.3)	0.9	0.4–2.2 (0.8)	7.5	0.8–73.1 (0.08)
HG2-GG3 versus HG1-GG1	5.6	3.7–8.7 (<0.001)	5.3	3.2–8.6 (<0.001)	3.9	2.3–6.8 (<0.001)	4.7	2.2–10.3 (<0.001)	14.1	1.8–110 (0.01)
HG3-GG1 versus HG1-GG1	3.8	1.9–7.5 (<0.001)	3.7	1.7–7.9 (<0.001)	2.8	1.2–6.4 (0.02)	3.9	1.5–10.0 (0.005)	22.6*	2.8–183* (0.003)
HG3-GG3 versus HG1-GG1	5.0	2.7–9.2 (<0.001)	5.1	3.1–8.5 (<0.001)	4.2	2.3–7.8 (<0.001)	6.0	2.3–15.4 (<0.001)	8.7	1.7–43.5 (0.008)

**Table 4:** Showing the correlation of scaled Schoenfeld residuals for each combination of histopathological and genomic grade with time for histopathological and genomic grade (rho = correlation coefficient, HG = histopathological grade, GG1 = low genomic grade, and GG3 = high genomic grade). A significant correlation shows time-dependent covariate effects. Negative correlation means that the prognostic effects get attenuated over time, and a positive correlation means that the prognostic effects get strengthened over time.

	Alldata		ER+ve		ER+ve Node −ve		ER+ve Stage I	
	rho	*P*	rho	*P*	rho	*P*	rho	*P*
RFS								
HG1-GG3	0.04	0.5	−0.07	0.3	−0.09	0.2	−0.09	0.4
HG2-GG1	0.11	0.6	−0.01	0.9	0.08	0.4	0.04	0.8
HG2-GG3	0.12	0.02	0.01	0.9	0.11	0.1	−0.21	0.2
HG3-GG1	− **0.18**	<0.001	−0.08	0.2	− **0.14**	0.007	−0.12	0.2
HG3-GG3	− **0.30**	<0.001	− **0.16**	0.05	− **0.22**	<0.001	− **0.23**	0.01

DMFS								
HG1-GG3	0.08	0.3	0.00	1	0.12	0.2	−0.08	0.6
HG2-GG1	−0.01	0.9	0.02	0.8	-0.05	0.7	−0.02	0.9
HG2-GG3	−0.03	0.7	−0.04	0.7	− **0.28**	0.02	− **0.38**	0.03
HG3-GG1	− **0.21**	<0.001	−0.09	0.3	− **0.17**	0.07	− **0.30**	0.09
HG3-GG3	−0**.29**	<0.001	− **0.17**	0.06	− **0.29**	0.003	− **0.39**	0.01

**Table 5:** Illustrating the time changing prognostic effects of histopathological grading. The table compares the hazard ratios of histopathological and genomic grading for the first seven years to the hazard ratios of the same from seven years onwards. This analysis was performed on the entire dataset.(HR = hazard ratio, HG = histopathological grade, GG1 = low genomic grade, and GG3 = high genomic grade).

	Recurrence-free survival		Distant metastasis-free survival	
	HR(0–7 yrs) (95% CI)	HR(7 yrs-EOS) (95% CI)	HR(0–7 yrs) (95% CI)	HR(7 yrs-EOS) (95% CI)
HG1-GG3 versus HG1-GG1	**10.0 **(4.0–24.9)	**1.4 **(0.2–9.0)	**26.5 **(4.7–149)	**1.1 **(0.2–5.6)
HG2-GG1 versus HG1-GG1	**4.6 **(1.9–11.4)	**1.5 **(0.8–2.6)	**9.6 **(1.4–66.2)	**0.9 **(0.4–2.1)
HG2-GG3 versus HG1-GG1	**11.0 **(5.3–22.7)	**1.8 **(0.9–3.8)	**32.1 **(5.1–202)	**1.2 **(0.3–4.3)
HG3-GG1 versus HG1-GG1	**7.4 **(3.6–14.8)	**0.5 **(0.1–3.9)	**22.7 **(3.6–144)	**0.4 **(0.05–3.7)
HG3-GG3 versus HG1-GG1	**10.3 **(5.3–20.1)	**0.7 **(0.3–1.9)	**31.6 **(5.6–179)	**0.5 **(0.2–1.3)
